# Advancing drug discovery through assay development: a survey of tool compounds within the human solute carrier superfamily

**DOI:** 10.3389/fphar.2024.1401599

**Published:** 2024-07-09

**Authors:** Daniela Digles, Alvaro Ingles-Prieto, Vojtech Dvorak, Tamara A. M. Mocking, Ulrich Goldmann, Andrea Garofoli, Evert J. Homan, Alberto Di Silvio, Lucia Azzollini, Francesca Sassone, Mario Fogazza, Felix Bärenz, Antje Pommereau, Yasmin Zuschlag, Jasper F. Ooms, Jeppe Tranberg-Jensen, Jesper S. Hansen, Josefina Stanka, Hubert J. Sijben, Helena Batoulis, Eckhard Bender, Riccardo Martini, Adriaan P. IJzerman, David B. Sauer, Laura H. Heitman, Vania Manolova, Juergen Reinhardt, Alexander Ehrmann, Philipp Leippe, Gerhard F. Ecker, Kilian V. M. Huber, Thomas Licher, Lia Scarabottolo, Tabea Wiedmer, Giulio Superti-Furga

**Affiliations:** ^1^ Department of Pharmaceutical Sciences, University of Vienna, Vienna, Austria; ^2^ CeMM Research Center for Molecular Medicine of the Austrian Academy of Sciences, Vienna, Austria; ^3^ Division of Drug Discovery and Safety, LACDR, Leiden University, Leiden, Netherlands; ^4^ Science for Life Laboratory, Department of Oncology-Pathology, Karolinska Institutet, Stockholm, Sweden; ^5^ Axxam SpA, Bresso, Italy; ^6^ Sanofi, Integrated Drug Discovery, Industriepark Hoechst, Frankfurt am Main, Hessen, Germany; ^7^ Centre for Medicines Discovery, Nuffield Department of Medicine, University of Oxford, Oxford, United Kingdom; ^8^ Lead Identification and Characterization, Bayer Pharmaceuticals, Wuppertal, Germany; ^9^ CSL Vifor, StGallen, Switzerland; ^10^ Novartis Pharma AG, Basel, Switzerland; ^11^ Center for Physiology and Pharmacology, Medical University of Vienna, Vienna, Austria

**Keywords:** solute carrier, transport protein, tool compound, KNIME, transport assay

## Abstract

With over 450 genes, solute carriers (SLCs) constitute the largest transporter superfamily responsible for the uptake and efflux of nutrients, metabolites, and xenobiotics in human cells. SLCs are associated with a wide variety of human diseases, including cancer, diabetes, and metabolic and neurological disorders. They represent an important therapeutic target class that remains only partly exploited as therapeutics that target SLCs are scarce. Additionally, many small molecules reported in the literature to target SLCs are poorly characterized. Both features may be due to the difficulty of developing SLC transport assays that fulfill the quality criteria for high-throughput screening. Here, we report one of the main limitations hampering assay development within the RESOLUTE consortium: the lack of a resource providing high-quality information on SLC tool compounds. To address this, we provide a systematic annotation of tool compounds targeting SLCs. We first provide an overview on RESOLUTE assays. Next, we present a list of SLC-targeting compounds collected from the literature and public databases; we found that most data sources lacked specificity data. Finally, we report on experimental tests of 19 selected compounds against a panel of 13 SLCs from seven different families. Except for a few inhibitors, which were active on unrelated SLCs, the tested inhibitors demonstrated high selectivity for their reported targets. To make this knowledge easily accessible to the scientific community, we created an interactive dashboard displaying the collected data in the RESOLUTE web portal (https://re-solute.eu). We anticipate that our open-access resources on assays and compounds will support the development of future drug discovery campaigns for SLCs.

## Introduction

Cells need to acquire the nutrients they require to grow, differentiate, and exert their functions. Molecular transporters play a critical role in this process, and their activity must be coordinated with metabolic needs and cellular processes. Solute carrier transporters (SLCs) constitute the largest superfamily of molecular transporters, with more than 455 members arranged into 66 families (Hediger et al., 2013; Ferrada and SupertiFurga, 2022). SLCs control nutrient uptake, ion transport, and waste removal, and hence, they are vital for maintaining metabolic homeostasis (Hediger et al., 2013; Meixner et al., 2020; Nguyen et al., 2021; Pizzagalli et al., 2021).

Importantly, SLCs also constitute paths for drug absorption into specific organs (Giacomini et al., 2010; Kell, 2016; Girardi et al., 2020), and genetic polymorphisms are associated with a plethora of human diseases, such as diabetes (Sladek et al., 2007; Dwivedi et al., 2019), cancer (ElGebali et al., 2013; Papalazarou and Maddocks, 2021), and neurological diseases (Nguyen et al., 2021; Schlessinger et al., 2023). There are also several rare diseases and inborn errors of metabolism, such as defects in thiamine and folate uptake, that are ascribed to SLCs (Zhao and Goldman, 2013). For this reason, SLC transporters have emerged as attractive targets for drug discovery, and several novel pharmacological approaches have been created to modulate their activity (Giacomini et al., 2010; Lin et al., 2015; Colas et al., 2016; Wang et al., 2020; Casiraghi et al., 2021; Sijben et al., 2022b; Dvorak and SupertiFurga, 2023; Gyimesi and Hediger, 2023; Schlessinger et al., 2023).

Despite their physiological and medical relevance, the development of drugs targeting SLCs is challenging, and only 22 SLCs are targeted by approved drugs (Wang et al., 2020; Kelleher et al., 2023), which is a disproportionally low number of therapeutics relative to other pharmacologically exploited protein families (Santos et al., 2017; Oprea et al., 2018). One of the main factors contributing to the successful development of drugs against other membrane proteins, such as GPCRs, ion channels, and kinases, is the abundance of robust, specific, and high-throughput functional assays for these protein families (Santos et al., 2017; Brown, 2023). In contrast, robust functional assays suited to SLCs were scarce until recently. This is in part because i) a large proportion of SLCs are still orphans in terms of substrate specificity (CésarRazquin et al., 2015; Meixner et al., 2020; Superti-Furga et al., 2020), ii) SLCs do not rely on secondary signaling molecules serving as robust assay readouts (Colas et al., 2016; Guan, 2022), and iii) systematic purification and reconstitution of human SLC proteins for *in vitro* assays remain challenging (Mancia and Love, 2010; Martin and Sawyer, 2019).

One of the goals of the RESOLUTE research consortium was the development of novel assays for SLC transporters (Superti-Furga et al., 2020; Dvorak et al., 2021; Wiedmer et al., 2022). In a recent review, the consortium described available assay technologies for SLC research (Dvorak et al., 2021). Three years after the publication of the review, the consortium partners have deployed and further developed several of the presented assay technologies. We have set-up 119 assays for 74 SLCs, and some of these assays have been published in original research articles (Sijben et al., 2021a; Sijben et al., 2021b; Sijben et al., 2022a; Mocking et al., 2022; Bongers et al., 2023; Pommereau et al., 2023) or in the open-access repositories Zenodo, PubChem, and ChEMBL.

In addition to the main obstacles for assay development as previously described, such as the lack of annotated substrates, electroneutrality, or intracellular localization (Dvorak et al., 2021), we realized that a major bottleneck was the lack of well-annotated, specific, and potent tool compounds. The availability of such compounds is very important when setting up an assay, especially for high-throughput screening pscreening purposes. On one hand, because specific inhibitors confirm that the signal recorded upon SLC stimulation is indeed mediated by the protein of interest since its presence reduces the signal in a dose-dependent manner. On the other hand, they can provide information about the inhibition efficacy. During the screening, the value obtained in the presence of a reference inhibitor is used for dual-normalization analysis, which is a more robust parameter and better allows inter-plate/inter-day comparisons. The lack of such control compounds frequently leads to problematic validation of functional assays, hindering the development of more drug-like compounds.

In this article, we give an overview of assays developed by RESOLUTE and highlight the urgent need for a resource that provides a good annotation for experimentally characterized SLC tool compounds. To address this need, we developed a KNIME workflow to integrate known inhibitors from different data sources and created a module with scored compounds targeting SLCs (https://re-solute.eu/resources/dashboards/toolcompounds). Last, we used some of our established assays to perform systematic tests with a small set of compounds across a panel of different SLCs to assess their selectivity. We anticipate that this SLC compound dashboard will assist with the development of transport assays through facilitating a simple and informed selection of SLC-targeting compounds.

## Results

### Development of assays for SLC transporters by the RESOLUTE consortium

The development of functional assays for SLC transporters has been an important objective of the RESOLUTE consortium and is key to unlocking this disease-relevant protein family for drug discovery. In a recent publication (Dvorak et al., 2021), we detailed the cell-based assay technologies used for transport assay development within the consortium. As a minimal criterion, an assay was considered validated if there was a significant difference in signal readout between an SLC-expressing cell line and control cells. Experimentally, for SLC-expressing cell lines, we normally used stably overexpressing cells lines, while for control cell lines, we used wild-type or knockout cell lines. For medium- (MTS) or high-throughput (HTS) assay acceptance, additional criteria had to be fulfilled, such as the stability over time, signal-to-noise ratio, inter- and intra-plate variability, robust Z’ factor, and reference substrate/inhibitor affinity determination and reproducibility. Signal readouts were typically monitored in a dose-dependent manner, following the acute addition of a substrate. However, for SLCs located in organellar membranes, such as mitochondrial carriers including SLC25A28 and SLC25A51, perturbations to acutely change substrate concentration changes are often not feasible. In these instances, we resorted to measure differences in steady-state signals instead.

**FIGURE 1:**
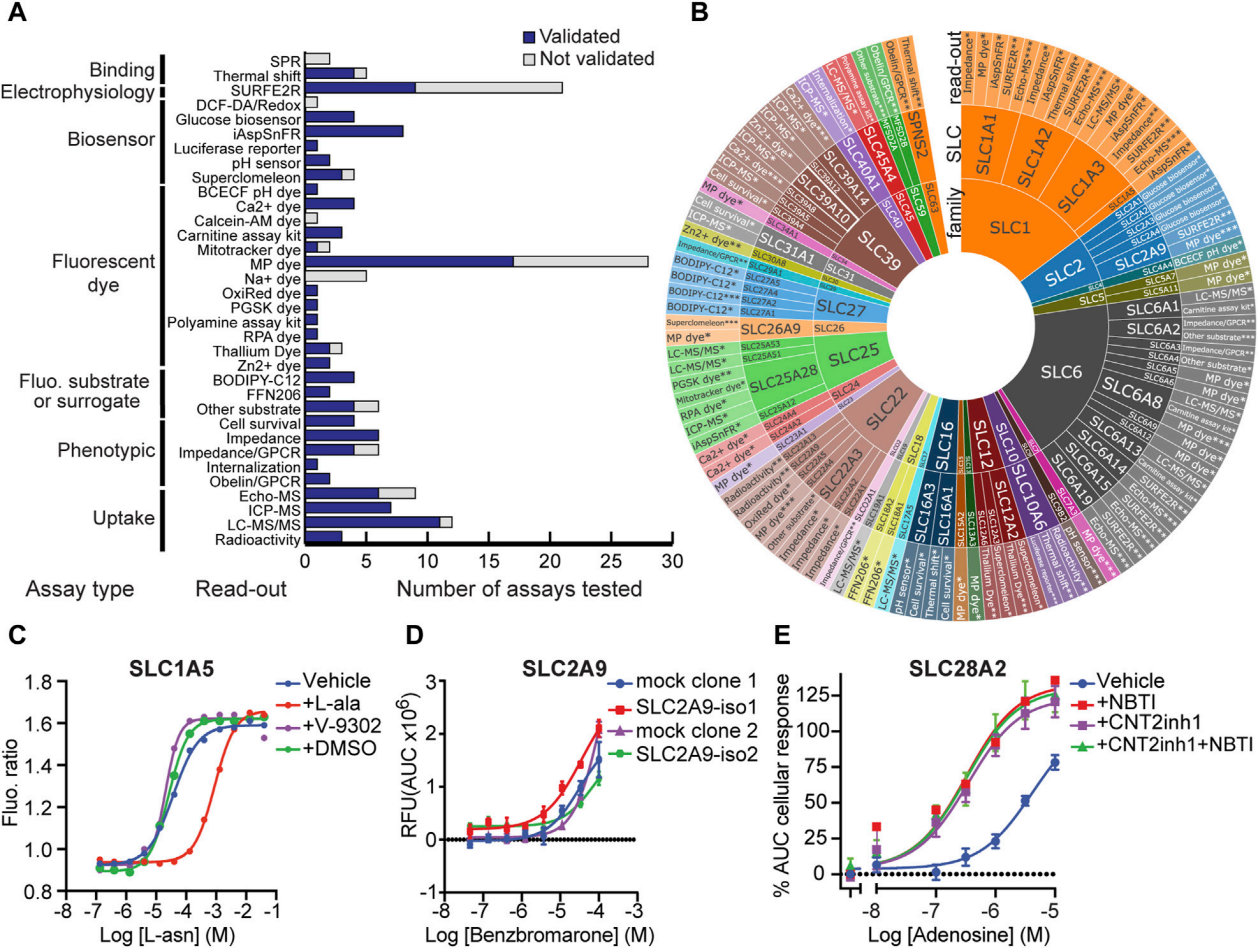
SLC functional assays developed in RESOLUTE. **(A)** Technologies tested by RESOLUTE. Different assay types and the assay readout are shown. The bar chart indicates the number of successfully developed assays in comparison to the total number of investigated assays. **(B)** Assays validated by RESOLUTE. Detailed assay readout used for each SLC is displayed for each SLC, classified by the SLC family. *Proof-of-concept, **medium-throughput, and ***high-throughput assays. **(C)** Measurement of asparagine uptake in the SLC1A5 assay after its conversion to aspartate by transfected gpASNase and using the biosensor AspSnFR HEK293 cells. To test the effect of the reported inhibitor V-9302, cells were pretreated with 50 µM of V-9302. **(D)** Dose–response curve of the reported inhibitor benzbromarone using a membrane potential dye-based assay on HEK293 cells stably expressing isoform-1 or -2 of SLC2A9, a comparison with two different mock cell line clones. **(E)** Effect of “CNT2 inhibitor 1” (CNT2inh1) on adenosine-mediated response in non-induced (-dox) HEK293–JumpIn–SLC28A2 cells using an impedance-based assay. Dose–response curves of adenosine with or without pre-treatment with the SLC29A1 inhibitor NBTI and/or SLC28A2 inhibitor “CNT2 inhibitor 1”. Cells were pretreated with 31.6 µM “CNT2 inhibitor 1” and/or 1 µM NBTI to inhibit SLC28A2 and SLC29A1, respectively, for 1 h prior to stimulation with increasing concentrations of adenosine. Data points show the mean ± SEM.

In total, RESOLUTE tested 163 assays (Figure 1A and Supplementary Table S1), each defined by a specific combination of an assay readout and an SLC, e.g., membrane potential (MP) dye assay applied to SLC1A3. Out of these, 119 assays for 74 different SLCs were successfully developed (“validated assays”). These SLCs span 31 out of the 66 known SLC families. Notably, this number includes 28 MTS and 19 HTS formats covering 29 SLCs (Figure 1B).

As shown in Figures 1A, B, MP dye-based assays account for the largest share of validated assays in RESOLUTE. MP dye assays assess substrate transport by electrogenic SLCs expressed on the plasma membrane. Due to their cost-effectiveness and adaptability to high-throughput formats, MP dye assays remain the mainstay in transport assay development. To investigate the possible reasons for assay failure (“not validated”) in more detail, we further examined the protein localization and electrogenicity. For most of the investigated SLCs, electrogenicity was previously reported in the literature for the target protein or an ortholog. For two SLCs where the assay set-up was successful (SLC5A11 and SLC22A4), we found no previous mention of electrogenicity in the literature. For SLC1A6 and SLC1A7, we found contradicting reports on electrogenicity (Arriza et al., 1997; Shigeri et al., 2001; Abousaab et al., 2016) and could not validate the MP dye assay. However, we observed a low expression of the SLC in the plasma membrane (data not shown). Therefore, we cannot conclude if the assay failed due to missing electrogenicity of the SLC.

Additionally, while developing assays to fulfill the validation criteria of our functional assays, the effect of several inhibitors reported in the literature could not be reproduced in our assays or showed unspecific/indirect effects. V-9302, a putative inhibitor for SLC1A5, showed no effect in our SLC1A5 asparagine uptake assay (Hellweg et al., 2024) (Figure 1C). Benzbromarone was reported to inhibit SLC2A9-mediated orotic acid transport (Pommereau, A. et al. in revision), but the same effect is also present in non-transduced cells (mock) (Figure 1D), suggesting an unspecific SLC2A9-independent effect. An unspecific response was observed in the TRACT assay on the concentrative nucleoside transporter SLC28A2 (CNT2), where the putative SLC28A2 inhibitor “CNT2 inhibitor 1” was not selective as it also inhibited SLC29A1 (Figure 1E and Supplementary Figure S1) (Tatani et al., 2015). In summary, these three examples underscore the necessity for accurate identification and validation of specific inhibitors in the study of SLC transporters, as evidenced by the non-specific effects observed with several reported inhibitors in our assays. These findings prompted us to embark on an effort to compile information on all available SLC modulators and annotate and score them. This should serve as a guide for future transporter research.

### Compilation and scoring of compounds targeting SLC transporters

**FIGURE 2:**
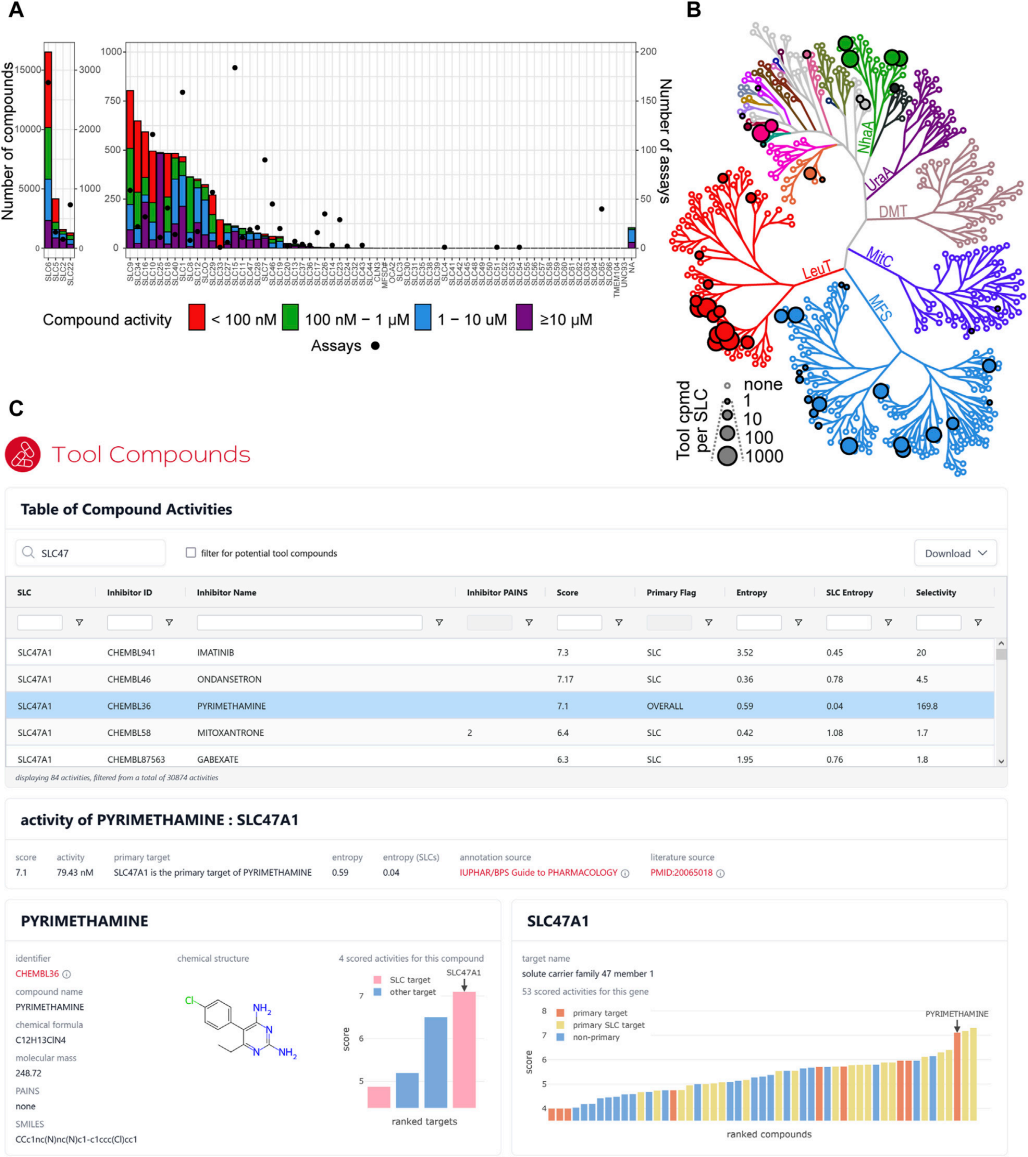
Tool compounds for SLCs. **(A)** Overview of compound (left Y-axis) and assay (right Y-axis) availability for each SLC family. Compound activity refers to the activity for its primary SLC target. **(B)** Number of potential tool compounds per SLC, visualized on an unrooted tree representation of a hierarchical clustering of similarities between structural models of SLCs. Each node represents an SLC, and its size corresponds to the number of described tool compounds. The nodes are colored by structural folds of the SLCs; MFS, major facilitator superfamily fold (MFS); LeuT, leucine transporter fold; MitC, mitochondrial carrier fold; DMT, drug/metabolite transporter fold; UraA, uracil transporter fold; NhaA, Na+/H+ antiporter fold. Clustering and fold classification as described in Ferrada and SupertiFurga (2022). **(C)** Interactive dashboard showing the table of all available compounds targeting SLCs filtered for SLC47 family genes. The activity of pyrimethamine targeting SLC47A1 is selected. This SLC is the primary target for this compound, but pyrimethamine is only the third most active compound for this SLC. All four scored target activities for pyrimethamine are displayed in the compound’s panel (lower left), and all 53 scored compound activities for SLC47A1 are displayed in the target gene’s panel (lower right).

To identify potent inhibitors targeting human SLCs, we generated the full inhibitor list by establishing an automated workflow to collect SLC inhibitor data from different databases (ChEMBL, GtoPdb, and PubChem) and integrate it with manually curated lists from RESOLUTE and EUbOPEN (https://www.eubopen.org/) consortia (Supplementary Table S2 and Figure 2A, see the Methods section for more details). We sought to provide literature references for each SLC–compound pair and at least one compound identifier. For compounds retrieved from chemical repositories, we reported dose–response inhibitory activity and selectivity (primary vs. secondary targets) of each compound toward SLCs. Following slightly adapted criteria from the EUbOPEN consortium (https://www.eubopen.org/chemogenomics), we shortlisted in the tool compound list only those compounds where the SLC of interest was reported as the primary target, which had reported IC_50_, EC_50_, Ki, or KD values ≤200 nM exhibiting at least 30-fold selectivity against other SLCs, and that contained no PAINS substructures (Figure 2B).

The full inhibitor list resulted in a list of 30,874 data rows, including 20,678 unique small molecules and 199 SLCs (Figure 2A). The overview of the physicochemical properties of all the compounds in the dataset shows that most compounds meet the Lipinski rules (Lipinski et al., 2001) (Supplementary Figure S2). Applying the rules for tool compounds to the initial list resulted in 6,876 inhibitors for 51 SLCs in total (Figure 2B). Of those, 5,390 compounds were tested against one transporter, 960 against two transporters, and 512 against three transporters (Table 1). Similarly, we found that a few compounds were tested more broadly against certain families. For instance, phloretin has the highest count of mentioned families with 10 different SLCs across seven families, imatinib against 9 SLCs from six different families, and DIDS against 16 SLCs across five families, while quinidine, ritonavir, and verapamil were tested against SLCs from five different families. Validating this workflow, we could find all eight SLC inhibitors reported at chemicalprobes.org (as of September 2023) (Antolin et al., 2023) in the full inhibitor list. Four of them were ranked first, and seven passed the criteria we set to be shortlisted in the tool compound list. A total of 530 out of 810 compound/SLC pairs retrieved in the manually curated lists were not reported in the investigated databases.

**TABLE 1:** Summary data from automatically collected activity values.

Total number of tested SLCs	Unique molecules	Unique molecules (tool compounds only)
1	14,014	5,390
2	3,160	960
3	3,157	512
4	71	11
5	26	1
6	10	1
7	7	1
8	2	0

Based on the full inhibitor list, we developed an interactive dashboard to allow for straightforward exploratory interactions with this dataset (Figure 2C). The dashboard shows all available compound activities for SLCs, which can be sorted and filtered to find either the compound or the SLC target of interest. Selection of an activity record leads to the display of additional details (entropy scores, source of annotation, etc.) and for the target SLC or the compound of interest (linked identifiers, chemical structure and formula, matched PAINS rules, etc.). In addition, interactive charts visualize all other compounds targeting the same SLC and all additional targets of the same compound, both ranked by activity. The dashboard is embedded in the RESOLUTE web portal and already freely available at https://re-solute.eu/resources/dashboards/toolcompounds. To the best of our knowledge, this is the most complete and up-to-date catalog of molecules targeting human SLC transporters.

**FIGURE 3:**
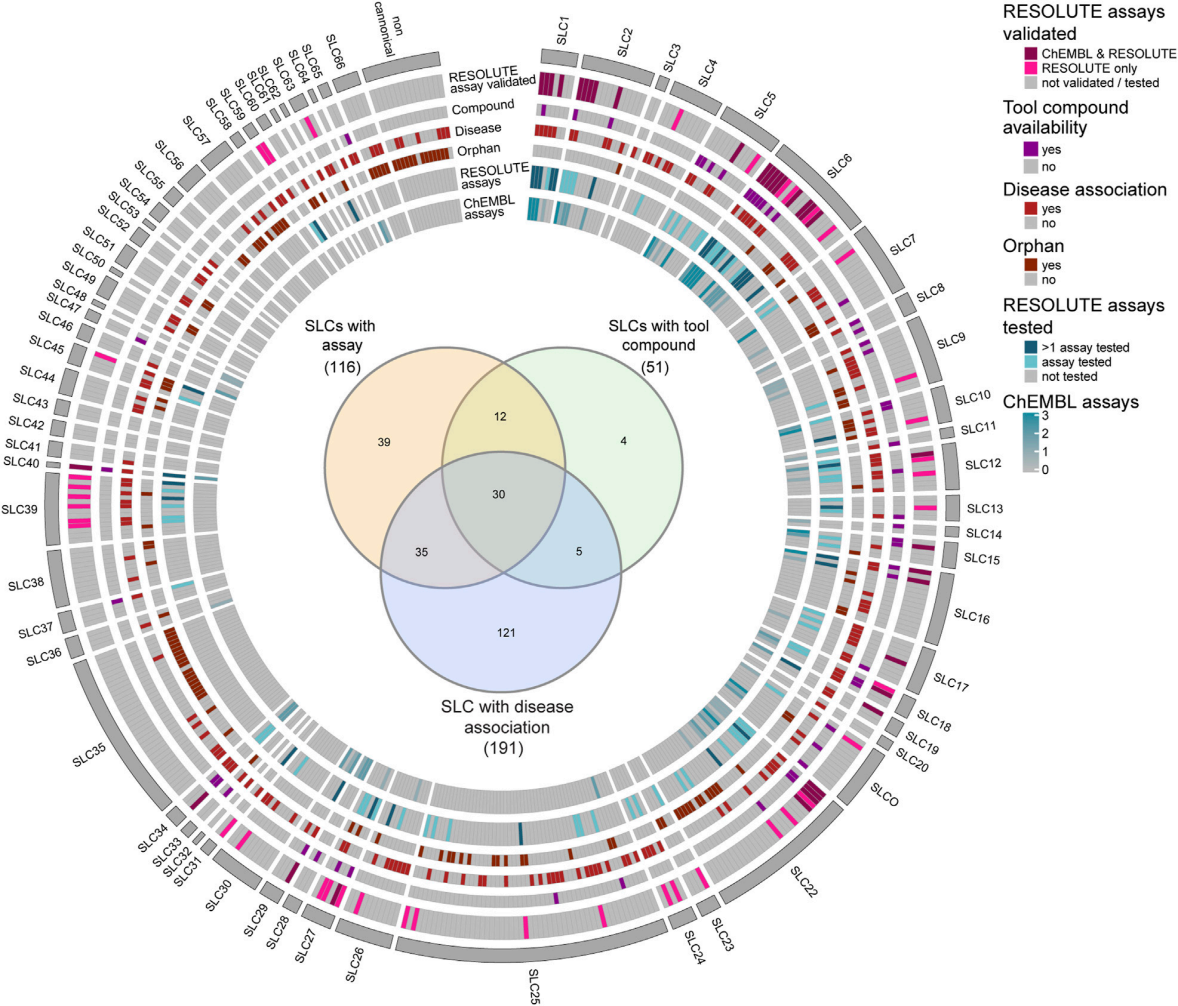
Schematic representation of features of SLC transporters, highlighting availability of assays, tool compounds, disease association, and orphan status. SLCs in the individual families are ordered numerically. The Venn diagram (Heberle et al., 2015) shows the intersection between SLCs with disease association, SLCs with available assays, and SLCs with available tool compounds. As a source for orphan status and disease association, the RESOLUTE knowledgebase was used (Goldmann et al., in preparation).

Next, to gain insights into the status of assay development and availability of tool compounds for the SLC class of transporters, we represented, in Figure 3, each individual SLC, showcasing the available assays, both developed by RESOLUTE and already documented in ChEMBL, as well as tool compounds identified in our workflow. We further highlighted the SLCs associated with human Mendelian diseases, as well as SLCs that remained orphan in terms of substrate (Goldmann et al., in preparation) (Supplementary Table S3). On one hand, we found a lack of assays and tool compounds for orphan SLCs. This is expected, given that a substrate is considered important to develop a transport assay (e.g., the SLC35 family). Yet this does not always prevent the possibility of assay development, such as in cases of phenotypic or binding assays (Figure 3 and Supplementary Table S3). RESOLUTE has developed assays for SLC targets that were not previously included in ChEMBL, as well as for targets that were already reported in this database. However, in these last cases, we focused on assay strategies that do not use radioactive ligands and assay formats feasible for high-throughput screening. On the other hand, we found that for many SLCs involved in disease, there is an assay available, but no tool compound was identified so far (Figure 3—inset). For this reason, these SLCs constitute attractive and feasible targets for compound screening campaigns (Supplementary Table S4).

### Systematic characterization of compounds in RESOLUTE assays

When investigating the available data for the 6,873 potential tool compounds, more than two-thirds of the molecules collected by our workflow had a selectivity entropy score of 0, indicating high selectivity (Uitdehaag and Zaman, 2011). However, most of them were, in fact, only tested against their respective target SLCs (Table 1). In such cases, selectivity cannot be claimed, as exemplified by the results obtained from our assay using the “CNT2 inhibitor 1” targeting SLC28A2 (Figure 1E).

Based on these findings, we started a pilot experiment to test a limited set of compounds against a panel of SLCs in RESOLUTE assays. We focused on inhibitors of the SLC1 (high-affinity glutamate and neutral amino acid transporter) and SLC6 families (neurotransmitters, amino acids, osmolytes, and energy metabolite transporters), giving preference to compounds compiled in the manually curated lists. To narrow down the number of compounds, we used several criteria, such as overall rank, activity, and selectivity for the primary SLC, as well as their availability from a trusted vendor. Based on these criteria, we selected 19 compounds, with eight reported to target the SLC1 family, eight targeting the SLC6 family, and three compounds targeting other SLC families (Supplementary Table S5 and Supplementary Figure S3).

The selected compounds were tested against 13 different SLCs in RESOLUTE assays (SLC1A1, SLC1A2, SLC1A3, SLC6A2, SLC6A4, SLC6A8, SLC6A9, SLC7A3, SLC9A1, SLC13A3, SLC22A3, and SLC23A1, SLC6A12). The results of active molecules are provided in Table 2, the full results are displayed in Supplementary Table S6, and the individual results are described in the following sections.

**TABLE 2:** Potencies of the screened reported and potential tool compounds. Only active values are shown; the full table including literature references can be found in the Supplementary Material. a) values were published previously (Sijben et al., 2021b); b) possibly due to an indirect effect of the assay/cell model; c) as reported in Supplementary Table S2.

Compound name	ChEMBL parent ID	SLC	Assay	Activity type	Activity value	Literature median activity^c^
DIDS	CHEMBL1162148	SLC1A1	EchoMS	pIC_50_	5.2	
TFB-TBOA	CHEMBL1257519	SLC1A1	EchoMS	pIC_50_	6.4	6.9
Riluzole	CHEMBL744	SLC1A1	EchoMS	pIC_50_	5.1	
WAY-213613	CHEMBL1628669	SLC1A1	EchoMS	pIC_50_	5.5	5.7
Loratadine	CHEMBL998	SLC1A1	EchoMS	pIC_50_	5.7	
KPH2f	CHEMBL5222644	SLC1A1	EchoMS	pIC_50_	6.0	
TFB-TBOA	CHEMBL1257519	SLC1A2	EchoMS	pIC_50_	8.1	7.9
Threo-beta-benzyloxyaspartic acid	CHEMBL475341	SLC1A2	EchoMS	pIC_50_	5.1	5.7
WAY-213613	CHEMBL1628669	SLC1A2	EchoMS	pIC_50_	6.6	7.1
TFB-TBOA	CHEMBL1257519	SLC1A3	EchoMS	pIC_50_	8.1	8.1
UCPH-101	CHEMBL474133	SLC1A3	EchoMS	pIC_50_	6.3	6.5
UCPH-102	CHEMBL1259233	SLC1A3	EchoMS	pIC_50_	6.8	6.4
Loratadine	CHEMBL998	SLC1A3	EchoMS	pIC_50_	5.2	
WAY-213613	CHEMBL1628669	SLC6A2	TRACT	pIC_50_	6	
Desipramine	CHEMBL72	SLC6A2	TRACT	pIC_50_	8.1^a^	8.7
Vanoxerine (GBR12909)	CHEMBL281594	SLC6A2	TRACT	pIC_50_	6.1^a^	6.9
KPH2f	CHEMBL5222644	SLC6A2	TRACT	pIC_50_	6	
Desipramine	CHEMBL72	SLC6A4	Uptake	pIC_50_	6.3	7.1
Vanoxerine (GBR12909)	CHEMBL281594	SLC6A4	Uptake	pIC_50_	5.7	6.8
DSP-1053	CHEMBL4226281	SLC6A4	Uptake	pIC_50_	7.2	8.8
BAY-876	CHEMBL4448899	SLC6A4	Uptake	pEC_50_	8.1^b^	
KPH2f	CHEMBL5222644	SLC6A4	Uptake	pIC_50_	6	
DSP-1053	CHEMBL4226281	SLC6A12	MP dye	Inhibition (3 µM) [%]	36	

**FIGURE 4:**
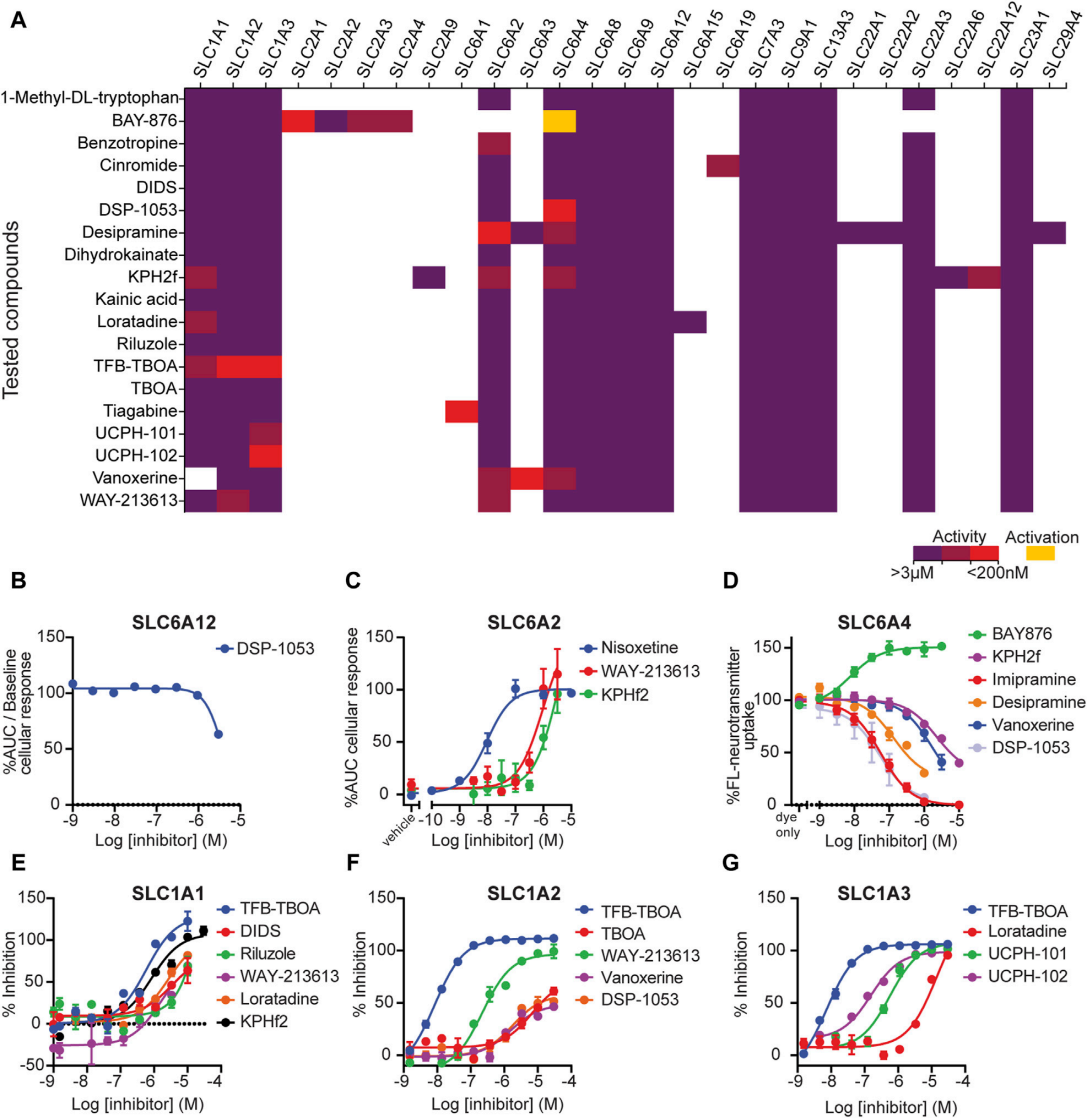
Tool compound testing. **(A)** Activity values for the compounds that were selected for testing combining data from public sources and in-house data. Violet: activity ≥ 3 μM; brown: 3 µM < activity <200 nM; red: activity ≤ 200 nM; and yellow: activator. **(B**–**G)** Dose–response curves of active compounds tested. **(B)** Concentration–inhibition curves of SLC inhibitors on dox-induced HEK293–JumpIn–SLC6A12 cells, as measured in a SLC6A12 membrane potential dye-based assay. **(C)** Concentration–inhibition curves of SLC inhibitors on dox-induced HEK293–JumpIn–SLC6A2 cells as measured in a TRACT assay. **(D)** Concentration–inhibition curves of SLC inhibitors on dox-induced HEK293–JumpIn–SLC6A4 cells, as measured in a fluorescent neurotransmitter uptake assay. **(E)** Concentration–inhibition curves of SLC inhibitors on dox-induced HEK293–JumpIn–SLC1A1 (EAAT3) cells, as measured in an EchoMS assay **(F)** Concentration–inhibition curves of SLC inhibitors on dox-induced HEK293–JumpIn–SLC1A2 (EAAT2) cells, as measured in an EchoMS assay. **(G)** Concentration–inhibition curves of SLC inhibitors on dox-induced HEK293–JumpIn–SLC1A3 (EAAT1) cells, as measured in an EchoMS assay. Data points show the mean ± SEM.

Overall, the previously reported inhibitory activities for the cognate target SLCs were confirmed, as summarized in Figure 4A. Although most inhibitors did not show off-target effects (Supplementary Table S6), we identified some cases in which we could detect additional off-target activities.

### DSP-1053

DSP-1053 was previously reported as an SLC6A4 (serotonin transporter) binder (Ki = 1.02 nM), serotonin uptake inhibitor (IC_50_ = 2.71 nM), and 5-HT1A (serotonin receptor) binder (Ki = 5.05 nM) (Yoshinaga et al., 2018). Although its selectivity against CYP2D6 was investigated in the original paper, so far, no data on selectivity against other SLCs could be found in the databases. In our assays, it displayed a modest 36% inhibition of SLC6A12 at 3 µM (Table 2). As it was inactive in all other tested SLCs (including the related neurotransmitter transporters), we could confirm its selectivity (Figure 4A).

### KPH2f

KPH2f (CHEMBL5222644) was previously reported as an SLC22A12 (URAT1) inhibitor with a dual-activity effect on SLC2A9 (GLUT9), showing IC_50_ values of 0.24 and 9.37 µM, respectively (Zhao et al., 2022). It was also tested against SLC22A6 (OAT1) and ABCG2 (IC_50_ = 32.14 and 26.74 µM). Although the requirement of an IC_50_ value below 200 nM was not met, the differences in potencies were large enough to result in a 30-fold selectivity, indicating this compound to be an interesting inhibitor. However, in our assays, we also found KPH2f to inhibit three more SLCs with IC_50_ values of approximately 1 μM: SLC1A1, SLC6A2, and SLC6A4 (Figures 4C–E). This indicated that this compound was not selective against SLC22A12. These off-targets not only belong to a different family than the reported target SLC22A12 but also have a different structural fold (Glt and MFS vs LeuT, respectively (Ferrada and Superti-Furga, 2022)).

### WAY-213613

The SLC1A2 inhibitor WAY-213613 was initially not meeting the selectivity criteria for a tool compound and can therefore only be found in the full inhibitor list (median pIC_50_ values of 7.1, 6.1, and 5.7 for SLC1A2, SLC1A3, and SLC1A1, respectively). However, as none of the potential tool compounds for this SLC were available for purchase, we included WAY-213613 in our assays. We could confirm the inhibitory activity against SLC1A2, but in our EchoMS assay, it showed a lower activity against SLC1A2 (pIC_50_ = 6.6, Figure 4F) than the one reported in the literature (Table 2). Furthermore, this compound was inactive against SLC1A3 (Supplementary Table S6) in the EchoMS assay. Finally, it was modestly inhibitory against SLC1A1 (pIC_50_ = 5.5, Figure 4E) and SLC6A2 (pIC50 = 6.0, Figure 4C) in our assays. Again, this SLC presents a different fold than the primary target of the inhibitor (LeuT for SLC6A2 and Glt for SLC1A2).

### BAY-876

An interesting and unexpected result was the increase in SERT (SLC6A4) activity by the selective SLC2A1 (GLUT1) inhibitor BAY-876 (Siebeneicher et al., 2016) (Figure 4D). Follow-up experiments showed that this effect is likely due to a change in the glucose concentration in the cell as the dose–response effect could not be reproduced under glucose-free conditions and the SERT activity seems to be generally higher in glucose-free conditions in HEK293–JumpIn–SLC6A4 cells (Supplementary Figure S4). This indicates that the apparent SERT activation could be mediated by inhibition of the HEK293 endogenous GLUT1.

## Discussion

The overarching goal of the RESOLUTE consortium was to work toward the “unlocking” of the large class of SLCs for drug discovery. Among many other activities to be reported elsewhere and listed in a previous paper (Superti-Furga et al., 2020), we developed several assays to allow for the potential identification of new chemical entities. We previously reported on cellular assays that we collected early in our campaign (Dvorak et al., 2021).

In this study, we provide an update on the various assays for SLCs developed by the RESOLUTE consortium while also presenting what we believe is the first systematic compilation of all SLC inhibitors reported in the literature. By including *in vitro* assays, we extend our previous report on cell-based transport assays for SLC transporters. As the practical use of assays assessing the biochemical activity of a protein is dependent on the availability of experimental compounds, we tested the assays developed by the RESOLUTE consortium with several of these SLC inhibitors.

Generally, we encountered several obstacles for cell-based SLC assay development, such as the difficulty to develop an assay in the absence of known substrates for the target transporter, electroneutrality of the target, or poor plasma membrane localization, among others (Dvorak et al., 2021). On the other hand, *in vitro* assays for SLCs require purified protein embedded in liposomes (Johnson and Lee, 2015; Drew et al., 2021) or membrane preparations (Bazzone and Barthmes, 2020; Bazzone et al., 2022; Pommereau et al., 2023). Although RESOLUTE has developed robust methods to express and purify SLCs for these purposes (Raturi et al., 2023), these approaches have a higher cost and labor requirement than cell-based approaches and require specialized know-how. Moreover, it remains difficult to control several parameters in *in vitro* assays, like directionality of transport or electrophysiology artefacts with substrates. Our assays were mostly optimized to fulfill throughput and robustness requirements for small-molecule screening campaigns and not pre-clinical drug development studies. Typical assays recommended by regulatory authorities to proceed with clinical trials rely on primary hepatocyte or CaCo cells, limiting their throughput. However, the assay formats developed in the RESOLUTE consortium may serve as an inspiration to extend the existing drug development assay arsenal with such medium-throughput assays in recombinant cells.

Irrespective of the kind of assay considered, a major constraint for successful assay development is the lack of well-annotated compounds able to modulate the activity of SLCs. In our investigation of compounds reported as selective inhibitors for SLC transporters, several were found to be either ineffective or non-selective against their purported target SLCs (Figures 1C–E). One plausible reason for this discrepancy could be that some SLC inhibitors were initially identified through screenings of FDA-approved drug libraries, which inherently already possess affinities for their cognate target proteins. For instance, some tyrosine kinase inhibitors (TKIs) have been documented to inhibit various transporters (Jouan et al., 2021; Xiu et al., 2023). Another factor contributing to this issue is the limited scope of selectivity testing. Typically, inhibitors in the literature are evaluated mainly for efficacy against the intended SLC and, only occasionally, one or two related SLCs for selectivity. Our collection of the SLC inhibitor data available from public databases showed that comprehensive characterization, like that performed for imipramine and ritonavir against eight SLCs each, remains rare (Supplementary Table S2). The situation reminds us of the early years of kinase inhibitor development, when in the absence of the ability of testing compounds kinome-wide, many drugs were later found to display dozens if not hundreds of additional targets (see (Reinecke et al., 2023) for a recent assessment).

In the process of compiling and scoring compounds targeting SLC transporters, we found a significant gap: more than 500 SLC/compound pairs from our manually curated lists were absent in existing databases. This discrepancy could partly stem from inconsistencies in chemical identifier nomenclature across different data sources (e.g., if different tautomeric forms were reported). Additionally, activity values reported as the percentage of inhibition are not retrieved by our automated workflow. A case to point out is the compound DIDS, which, although unlisted in the databases, was identified through our manual literature review as an inhibitor of 16 SLCs across five families for consistency to previous statement.

Even with our compilation of SLC inhibitors, selecting a potential tool compound to be used for assay development is not a straightforward task. We have compiled some parameters that can be considered, such as the potency against the SLC of interest (should be high), the selectivity entropy score (should be low), and the total number of tested SLCs (should be high). Pareto ranking could be used to identify the best-ranked inhibitor (see details on KNIME nodes used in the Methods section), but this might be biased and tends to select several inhibitors for the first rank. For a few SLCs, at least a thousand potential tool compounds are available (Figure 2B). Here, the list could be further limited by investigating the selectivity against other non-SLC targets as well as these might cause indirect effects in the assays. In cases where no potential tool compound is reported, it might still be worthwhile to investigate the full list of inhibitors to identify inhibitors that do not fulfill the criteria of a tool compound but could be optimized to do so. In any case, it is recommended to check the literature provided to see if additional data (e.g., percent inhibition) are available for the selected compound. Lists of tool compounds that meet specific criteria are collected in portals such as www.chemicalprobes.org and www.thesgc.org/chemical-probes. Although the number of validated tool compounds reported in these portals is still very small, it is worthwhile to check the SLC of interest there as these databases are periodically updated.

In our effort to improve the situation of available tool compounds, we tested a set of 19 compounds against 13 SLCs. Several of the tested inhibitors were identified to not meet our criteria as tool compounds. For KPH2f and loratadine, we did not test the original targets but could identify selectivity issues in our assays. For WAY-213613, we could reproduce the activity for the main target but found a selectivity issue as well. For some of the compounds, we did not find a statement of potency in the databases or the compound’s reported potencies were in the range of our tested maximum concentration. In those cases, we could not provide new insights on their usefulness as tool compounds: 1-methyl-DL-tryptophan, benzotropine, dihydrokainate, kainate, and riluzole. Other compounds were not selective based on the literature, but we could not identify any new off-target effects: TFB-TBOA, DIDS, threo-beta-benzyloxyaspartic acid, and vanoxerine (GBR12909). Again, some of the potencies were in the range of the tested maximum concentration.

We could also confirm the potencies and selectivity of several inhibitors, suggesting their usefulness as tool compounds: desipramine for SLC6A2, DSP-1053 for SLC6A4 (although both show side effects on the respective serotonin receptors), and UCPH-101 and UCPH-102 for SLC1A3. However, in the last two cases, the activity is above the 200 nM threshold. For some, we did not test the inhibitor against the main SLC target but could confirm the selectivity: BAY-876 for SLC2A1, tiagabine for SLC6A1, and cinromide for SLC6A19. The latter does not meet the 200 nM criterion but, according to our inhibitor collection, is the most potent inhibitor for SLC6A19 available.

Although we could confirm the inhibitory selectivity of the selective SLC2A1 inhibitor BAY-876 (Siebeneicher et al., 2016) and, therefore, its usefulness as a tool compound, we could observe an increased activation of SLC6A4 while testing this compound. This result is very likely an indirect effect as the dose–response effect could not be seen under glucose-free conditions (Supplementary Figure S4). It has been shown previously that SERT activity is dependent on glucose levels (Gonçalves et al., 2008), where a short-term exposure of Caco-2 cells to high glucose levels decreased SERT activity, while a long-term exposure increased SERT activity, without changing the affinity to serotonin significantly. The molecular mechanism of this phenomenon is not understood, and we speculate that in the used cell line (HEK293–JumpIn–SLC6A4), the activity of the serotonin transporter may be increased when glucose levels in the cells are reduced, either by inhibition of a glucose transporter or by removal of glucose from the media. Although the effects of serotonin and its transporters on cellular metabolism have been reported (Yabut et al., 2019; Suchacki et al., 2023), further studies are needed to explain this phenomenon in more detail, e.g., if the serotonin transporter is upregulated under low-glucose levels. These results point toward effects due to indirect effects or artefacts of the assay or cell model used to evaluate this target. This outcome highlights the importance of screening inhibitors against unrelated SLCs and alternative assays to fully characterize them in terms of potency and selectivity. Furthermore, this approach could also contribute to the mechanistic understanding of the SLC function.

Generally, we acknowledge that the data generated in this work are restricted to a set of 13 SLCs, albeit across seven SLC families. However, we consider this effort a blueprint for future public–private consortia to expand the charting of the functional and pharmacological landscape of human SLCs. Our work already combines resources and expertise from two EU-funded IMI consortia, RESOLUTE and EUbOPEN, indicating the magnitude and resources of this endeavor. We hope that our “pilot” study will spark further interest in the discussion about how to best annotate and characterize tool compounds and consequently the need for focused collaborative efforts between industry and academia. It is also worth mentioning that for making the tool compounds listed in this article useful for *in vivo* studies, a much broader pharmacokinetic characterization would be required.

In summary, to help with future SLC assays and drug development, we provide here a dashboard with available tool compounds (https://re-solute.eu/resources/dashboards/toolcompounds), as well as an updated transport assay collection (Supplementary Table S1). We anticipate that the resources and lessons learned described in this article will allow the development of novel transport assays and the identification of potent and specific tool compounds targeting SLCs, which will pave the way to unlock this superfamily of proteins in drug discovery.

## Materials and methods

### Compilation and scoring of potential tool compounds for SLCs

To compile a list of inhibitors, including recommendations on potential tool compounds, a KNIME workflow was implemented with KNIME version 4.7.4 (Berthold et al., 2009). The workflow collects and combines data on SLC inhibitors from different data sources, calculates selectivity measures, and provides a ranking and filters for potential tool compounds. Data shown in this paper were retrieved in August 2023.

Data from the ChEMBL database (version 33) (Mendez et al., 2019) were downloaded as an SQLite file and read into the KNIME workflow with SQLite Connector and DB Query Reader nodes. Assay data were retrieved for human proteins only, with an assay confidence score of at least seven, and without any data validity comment. To avoid duplicates, data points were excluded if flagged by the ChEMBL team as potential duplicates or if data originated from PubChem (src_id 7) or RESOLUTE (src_id 58). Values with µM units were converted to nM, and only data points with standard activity types of IC_50_, Ki, EC_50_, or Kd with nM units were retained. The activity values were transformed into the negative logarithm of the molar value. For the molecules, parent structures and ChEMBL IDs were retrieved for standardized structures (e.g., salts removed). Gene and protein names for human proteins with the status “reviewed” were downloaded from UniProt (UniProt Consortium, 2023) and joined with the ChEMBL targets via the UniProt accession number.

To include bioactivity data from the IUPHAR/BPS Guide to Pharmacology database (GtoPdb) version 2023.2 (Harding et al., 2022), the interactions data file from Harding et al. (2022) was downloaded, and only data for human SLCs were retained. In cases where a range of activity values was given, the average was calculated. To integrate GtoPdb with other data sources, PubChem substance identifiers were used to retrieve PubChem compound identifiers via the PubChem Power User Gateway (PUG) (Kim et al., 2018). From the CIDs, ChEMBL compound IDs were retrieved via UniChem (Chambers et al., 2013), or in case they were missing there, from PubChem. ChEMBL parent IDs were retrieved via the ChEMBL API (Davies et al., 2015). For cases where no ChEMBL IDs could be retrieved at all, the PubChem CIDs were used instead.

The third data source for bioactivity data was PubChem. PubChem Assay IDs (AIDs) were retrieved from PubChem PUG for a list of SLC Entrez Gene IDs provided by the RESOLUTE consortium. To reduce the overlap with other data sources, assays from sources “ChEMBL” and “IUPHAR-DB” were removed. Bioactivities were retrieved for the remaining AIDs, and only data points with non-missing activity values were retained. As described for GtoPdb, parent ChEMBL compound IDs were retrieved from PubChem CIDs where possible.

Data from ChEMBL on any reviewed human protein and data on SLCs from the other two sources were concatenated. Data points with activity types other than pEC_50_, pKd, pIC_50_, and pKi or empty activity values were removed. As ChEMBL also includes data for mutated proteins, which could lead to unwanted results when analyzing the data, data points with *mutant* in the assay description were removed (* is any number of symbols). If data points were duplicates according to activity type, activity value, parent ChEMBL compound ID, SMILES, gene name, and UniProt accession number, only one row was kept. Then, for each pair of ChEMBL compound ID and UniProt accession number, the median activity value was calculated. Compounds that were not tested on at least one SLC were discarded.

To assess the selectivity, a selectivity entropy score (Uitdehaag and Zaman, 2011) was implemented in KNIME. The entropy score was calculated twice for each compound, first against all SLCs (S_SLCs) and then against all proteins (S_other). Ideally, a low entropy value indicates a high selectivity of the inhibitor. However, as the inhibitors were not tested on a full panel of targets, but often only against one to three proteins, a low entropy value might not reflect its true selectivity. Therefore, Pareto ranking was performed using the corresponding node from the Erlwood KNIME open-source cheminformatics KNIME community extension. This ranking minimizes S_SLCs and S_other while maximizing the activity value and the count of SLCs the inhibitor was tested on.

The RESOLUTE list of SLC compounds was retrieved, as described previously (Meixner et al., 2020), and manually curated. The EUbOPEN list of SLC compounds was compiled as part of its chemogenomics searches (https://www.eubopen.org/chemogenomics), then manually curated to find commercially available compounds from trusted vendors (i.e., the suppliers with whom EUbOPEN have an agreement and whom they trust to be selling publicly available compounds), and then matched to basic theoretical criteria for being a chemogenomics compound.

To ensure that the tool compounds were on our list, tool compounds from chemicalprobes.org (Antolin et al., 2023) (filtered for protein family “transporter”) were downloaded (last accessed August 2023) and joined to our list using the compound ChEMBL identifiers and the SLC names.

To identify pan-assay interference compounds (PAINS) (Baell and Holloway, 2010), SMARTS patterns reported by Greg Landrum (http://rdkit.blogspot.com/2015/08/curating-pains-filters.html) which improved a SMARTS implementation reported earlier (Saubern et al., 2011) were included as substructure filters in the workflow.

To identify potential tool compounds, flags were calculated at different points in the workflow that can be used as filters in the full inhibitor list (Supplementary Table S2). A list of all available flags with their description can be found in the column explanation tab of Supplementary Table S2.

### Selection and distribution of inhibitors and SLCs for the pilot test in assays

For the final selection of compounds to test, the full list of inhibitors was filtered for compounds that could be purchased, which drastically reduced the number of data rows from over 30,000 to 2,237. Vendor information was compiled from the Probes and Drugs database version 04.2022 by combining subsets of ligands available from AdooQ, Axon Medchem, Cayman Chemical, Enamine, Mcule, MedChemExpress, Selleckchem, TargetMol, Tocris Bioscience, and ZINC. The selection focused on inhibitors for SLC families one and six to take advantage of the large number of reported potent inhibitors, as well as the assays that were available in the consortium. The inhibitors were selected individually not only based on rank criteria but also on whether they were mentioned in one of the manually curated lists by RESOLUTE and EUbOPEN. In addition, DIDS was selected, which appears to be an inhibitor of several SLCs from four different families but without dose-response values in the investigated databases. To expand the breadth of SLC families tested and further explore family selectivity, we selected two compounds targeting other SLC families: BAY-876, a well-known tool compound for SLC2A1, and KPH2f, a compound reported to target SLC22A12 and SLC2A9. Available data on the selected compounds can be found in Supplementary Table S6. The selected inhibitors were compiled by one partner from different vendors (Supplementary Table S5) and then distributed among the consortium members. Once the inhibitors were distributed among the partners, we selected SLCs based on the best performing assays across all families generated by RESOLUTE. The key criteria for us were a) assay reproducibility and b) coverage across distinct SLC families.

### Cell line generation

The Jump In T-REx human embryonic kidney 293 cell line (HEK293) expressing doxycycline-inducible human wild-type SLCs was generated by RESOLUTE, as described previously (Polesel et al., 2023). After thawing, HEK293 cells stably expressing all constructs were selected in DMEM medium supplemented with 10% FBS, 5% penicillin/streptomycin, 0.0005% blasticidin, and 4% geneticin for 1 week.

### AspSnFR biosensor assay

HEK293 cells expressing AspSnFR were transiently transfected with EBPF-gpASNase1, trypsinized, and resuspended in 10% dialyzed FCS/PBS. Post-treatment with test compounds or vehicle, varying concentrations of asparagine were added. After a 30-min incubation at room temperature, flow cytometry analysis was performed using the BD LSR Fortessa X-20, following the protocol previously described (Hellweg et al., 2024).

### Membrane potential dye-based assay

FLIPR Membrane Potential Blue Dye was used to detect the compound activities for those membrane transporters that are electrogenic or whose activity is associated with changes in membrane potential, as previously described (Jensen and BräunerOsborne, 2004). In brief, cells are seeded at cell line-specific density in black clear-bottom poly-D-Lysine-coated 384-well plates (Greiner Bio-One) in DMEM high-glucose medium with 10% FBS, 1% P/S, and 2 mM UltraGlutamine I (25 µL/well). Furthermore, 24 h after seeding, the culture medium is removed, and the cells are loaded with 20 µL/well of 0.5 X FMP Blue Dye in assay buffer (modified Tyrode’s buffer, 130 mM NaCl, 5 mM KCl, 1 mM MgCl2, 2 mM CaCl2, 5 mM NaHCO3, and 20 mM HEPES pH 7.4). The plates are subsequently incubated for at least 30 min at room temperature in the dark. Then, 10 µL/well of test compounds and controls is added at defined concentrations (0.5% DMSO final conc.) in the FLIPR TETRA instrument (Molecular Devices), and emitted fluorescence is recorded using a λexc 510–545-nm/λem 565–625-nm filter. The dose–response curves for the 19 compounds were prepared starting from 3 µM (final top concentration), the half-log serial dilution factor. For data analysis, Genedata Screener 19.0 software was used. The response value as AUC/baseline was calculated, and the response value *versus* minimum signal (blank controls) and maximum signal (neutral controls) wells was normalized to obtain a % activity according to the following formula:

$$\%Activity=100*\left(\frac{x-\;\langle \mathrm{MIN}\rangle }{\langle \mathrm{MAX}\rangle -\;\langle \mathrm{MIN}\rangle }\right).$$

The robust Z prime factor (RZ’ factor) was calculated with the following formula:

$$RZ^{\prime }=1-\frac{3*\left({RSD}_{\mathrm{MAX}}+{RSD}_{\mathrm{MIN}}\right)}{Abs\left(\langle \mathrm{MAX}\rangle \;-\;\langle \mathrm{MIN}\rangle \right)},$$

where the RZ’ factor is based on the same formula as the Z′ factor (Zhang et al., 1999) but standard deviations and means are replaced by the robust standard deviations and medians, respectively. Curve fitting profiles were performed on each dose–response curve with the analyzer module of Genedata Screener 19.0 on % activity.

### Impedance-based MPP + transport assays

The impedance-based transport assay for SLC22A3 (OCT3) using MPP+ as a substrate was performed as previously described (Mocking et al., 2022). In short, HEK293–JumpIn–OCT3 (SL22A3) cells were seeded (60.000 cells/well) on an E-plate with 1 μg/mL doxycycline and grown for 22 h using the xCELLigence real-time cell analyzer (RTCA) SP or MP system. After 22 h, the cells were pretreated with 10 µM or concentrations of inhibitor ranging from 10^–5^ M to 10^–9^ M in semi-log steps with vehicle (PBS +0.1% DMSO) or corticosterone (control) for 1 h prior to stimulation with 100 µM MPP+. Changes in cellular morphology were monitored for a total of 2 h.

### Impedance-based TRACT assay

The impedance-based TRACT assay using the xCELLigence system for the norepinephrine transporter (NET) was performed and analyzed as previously described (Sijben et al., 2021b; Bongers et al., 2023). In brief, HEK293–JumpIn–NET (SLC6A2) or HEK293–JumpIn–CNT2 (SLC28A2) cells were seeded (60.000 cells/well) on an E-plate with 1 μg/mL doxycycline, and cell growth was monitored for 22 h using the xCELLigence real-time cell analyzer (RTCA) SP or MP system. For NET after 22 h, the cells were pretreated with 10 µM or concentrations of the inhibitor ranging from 10–5 M to 10–9 M in semi-log steps with a vehicle (PBS +0.1% DMSO) or nisoxetine (control) for 1 h prior to stimulation with 1 µM norepinephrine. Immediately after stimulation, changes in cellular response were monitored every 15 s for a total of 30 min. For SLC28A2, TRACT assay was performed similar to NET utilizing the activity of endogenous adenosine A2B receptors. HEK293–JumpIn–SLC28A2 cells were pretreated with 31.6 µM CNT2 inhibitor 1 or 1 µM NBTI for 1 h to inhibit SLC28A2 and SLC29A1, respectively. Cells were stimulated with increasing concentrations of adenosine, and the response was monitored for 60 min.

### Displacement assay

Displacement assay with [3H]NBTI on human erythrocyte membranes expressing ENT1 (SLC29A1) was performed at 25°C, as described previously (Vlachodimou et al., 2020).

### Fluorescent uptake assay

HEK293–JumpIn–SERT (SLC6A4) cells were regularly maintained in DMEM supplemented with 10% dialyzed FCS, 2 mM Glutamax, 100 IU/mL penicillin, and 100 μg/mL streptomycin. HEK293–JumpIn–SERT cells were seeded (60,000 cells/well) in a poly-D-lysine-coated black clear-bottom 96-well plate and grown for 24 h in the presence of 1 μg/mL doxycycline to induce SERT expression. The cells were incubated with concentrations of inhibitor ranging from 10^–5^ M to 10^–9^ M in semi-log steps with a vehicle or imipramine (control) in assay buffer (HBSS +20 mM HEPES) for 1 h prior to the initiation of the uptake by the addition of a fluorescent neurotransmitter dye (Molecular Devices). The uptake was measured every 25 s for 1 h using a FlexStation 3 multi-plate reader (Molecular Devices). Data were analyzed by calculating the area under the curve (AUC) and subtracting basal fluorescence. Data were converted to the percentage fluorescent neurotransmitter uptake, whereby the dye-only condition was set at 100% and full inhibition with 10 µM imipramine was set to 0%.

### BCECF-AM pH recovery assay

MDA-MB-468 breast cancer cells, which exhibit elevated NHE1 activity (SLC9A1) (Amith et al., 2016; Li and Fliegel, 2022), were used for the measurement of intracellular pH. The cells were seeded (6.000 cells/well) in poly-d-lysine-coated 384-well plates (black, transparent bottom) and grown for 24 h at 37°C and 5% CO_2_. The media was removed, and the cells were washed once in sodium-free loading buffer (115 mM choline chloride, 20 mM NH_4_Cl, 5 mM KCl, 1 mM CaCl_2_, 1 mM MgCl_2_, 20 mM HEPES pH 7.4, and 5 mM glucose) and subsequently incubated for 30–45 min at 37°C in a loading buffer containing 10 µM 2′,7′-Bis(2-carboxyethyl)-5(6)-carboxyfluorescein tetrakis (acetoxymethyl) ester (BCECF-AM). The loading buffer was removed, and the cells were washed twice with NH_4_Cl-free acidification buffer (133.8 mM choline chloride, 4.7 mM KCl, 1.25 mM CaCl_2_, 1.25 mM MgCl_2_, 0.97 mM K_2_HPO_4_, 0.23 mM KH_2_PO_4_, 5 mM HEPES pH 7.4, and 5 mM glucose) using a plate dispenser (Multidrop Combi+, Thermo Fisher Scientific, US). The cells were incubated 5 min with 15 µL compounds at 2× concentration in acidification buffer. The assay readout was acquired using a plate reader (POLARstar Omega, BMG LABTECH, Germany) set to measure Ex./Em. at 440 nm/520 nm and 485 nm/520 nm over a period of 15 min. A baseline read was recorded before the plate readers automated dispensing of 15 µL recovery buffer (133.8 mM NaCl, 4.7 mM KCl, 1.25 mM CaCl_2_, 1.25 mM MgCl_2_, 0.97 mM Na_2_HPO_4_, 0.23 mM NaH_2_PO_4_, 5 mM HEPES pH 7.4, and 5 mM glucose), which initializes cellular pH recovery. The mean baseline measurement was subtracted from the intensities of each well, and a fluorescence intensity ratio for excitations at 485 nm and 440 nm was calculated. The data were normalized to DMSO (maximum) and no recovery controls (minimum). The data were analyzed using R, and the dr4pl package was used to fit dose–response data to the four-parameter logistic model.

### Echo-MS assay

Before the Echo-MS experiment, 15,000 cells per well were seeded into the wells of a 384-well plate (Beckman Coulter) and induced with 1 μg/μL doxycycline. Each 384-well assay plate contained a low control (1% DMSO in wash buffer on the wild-type cell line HEK293; column 1) and a high control (1% DMSO in wash buffer on cells overexpressing SLCs; columns 2–4) to assess compound-related inhibition of the transporter activity. The next day, compounds with stock solutions of 10 mM were first diluted to a final concentration of 90 µM before starting a 10-stage dilution series with a dilution factor of 1:3 with the CyBio FeliX pipetting robot. After the supernatant in the plate of the cell plate assay was discarded (BlueWasher, BlueCatBio), 10 µL of potassium/wash buffer including the compound dilution series was transferred by the pipetting robot (FeliX, CyBio), yielding a highest final concentration of 30 µM compound per well. After an incubation time of 15 min, 20 μL of L-Glutamic acid-13C5,15N with a concentration of 300 µM was added and incubated in an incubator at 37°C, 7.5% CO_2_, and 95% humidity. After 1 h incubation time, the wells were washed with 70 µL potassium buffer per well, and the supernatant was discarded before the precipitation process was started by adding 90 µL/well precipitation reagent (60% MeOH, 29% H_2_O, 11% formic acid) using a Multidrop Combi reagent dispenser (Thermo Fisher Scientific). After 30 min on the shaker at 500 rpm, the plates were centrifuged for 20 min at 2,000 rpm. Finally, 40 µL/well was transferred on qualified 384-well plates and then measured with Echo-MS/ADE-MS (Sciex). ADE-MS measurements were performed on an ADE-MS device (Sciex) operated with Sciex OS software (version 2.1.6.59781). A carrier solvent (60% acetonitrile, 39% H_2_O, and 1% formic acid) was used at a constant flow rate of 300 μL/min. For all samples, an ejection volume of 5 nL (2 droplets) and a repetition rate of 2.5 s/well were used. Triple Quad 6500+ was operated in a positive mode using the following settings: vaporizer temperature 450°C, spray voltage 5500 V, ion source gas 1: 90 psi, ion source gas 2: 70 psi, curtain gas 20 psi, CAD gas 9 psi, dwell time 95 m, DP 35 V, EP 10 V, CE 14 V, and CXP 18 V. Data were processed with Sciex OS and further processed with Genedata Screener (version 19.0.9-Standard). Transporter inhibition by the compounds was tracked by analyzing the MS signal area values of uptaken labeled glutamic acid into the cell. The signal ratio of the reaction product to the respective standard was calculated to diminish variations ascribed to the sample preparation procedure or the MS readout. Average control values were calculated and set to 100% activity (high controls) and 0% activity (low controls), while the response values of compound-containing wells were normalized against the controls and expressed as the percentage of control (PoC).

## Data Availability

Assays presented in this study are listed in the article Supplementary Material; referencing entries in ChEMBL (RRID:SCR_014042l; DOI:10.25504/FAIRsharing.m3jtpg) and PubChem (RRID:SCR_004284; DOI:10.25504/FAIRsharing.qt3w7z) repositories. The compiled assay compound activities are listed in the article/Supplementary Material; (accession numbers in "chembl_id" and "act_data_source_ID" columns), referencing entries in ChEMBL (RRID:SCR_014042l; DOI:10.25504/FAIRsharing.m3jtpg), PubChem (RRID:SCR_004284; DOI:10.25504/FAIRsharing.qt3w7z) and GtoPdb (RRID:SCR_013077; DOI:10.25504/FAIRsharing.f1dv0) repositories.
