# Supplementary material for: Advancing drug discovery through assay development: a survey of tool compounds within the human solute carrier superfamily
Source: Front Pharmacol. 2024 Jul 9;15:1401599. doi: 10.3389/fphar.2024.1401599 (PMC11267547; doi:10.3389/fphar.2024.1401599)
Supplement: Supplementary file 5 [file Table5.docx]

Supplementary Material

# Supplementary Figures

#
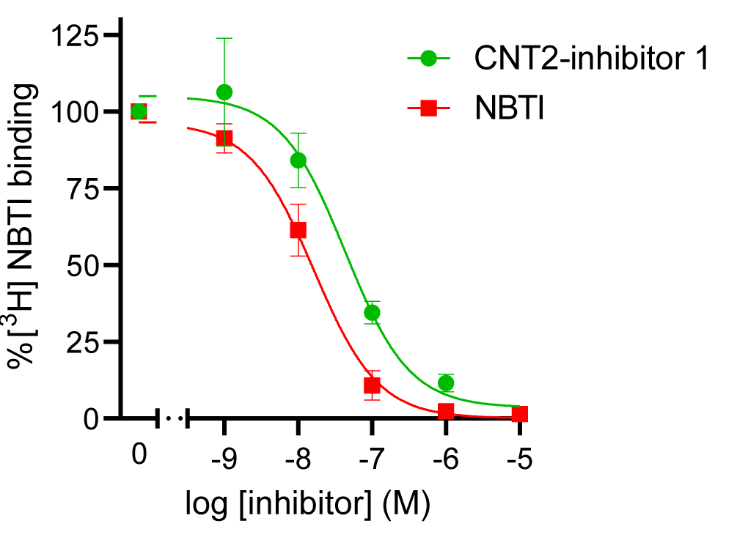


# Supplementary Figure 1. Displacement curves of [^3^H]NBTI by CNT2-inhibtor 1 and unlabeled NBTI from SLC29A1 endogenously expressed on erythrocyte membranes.

#
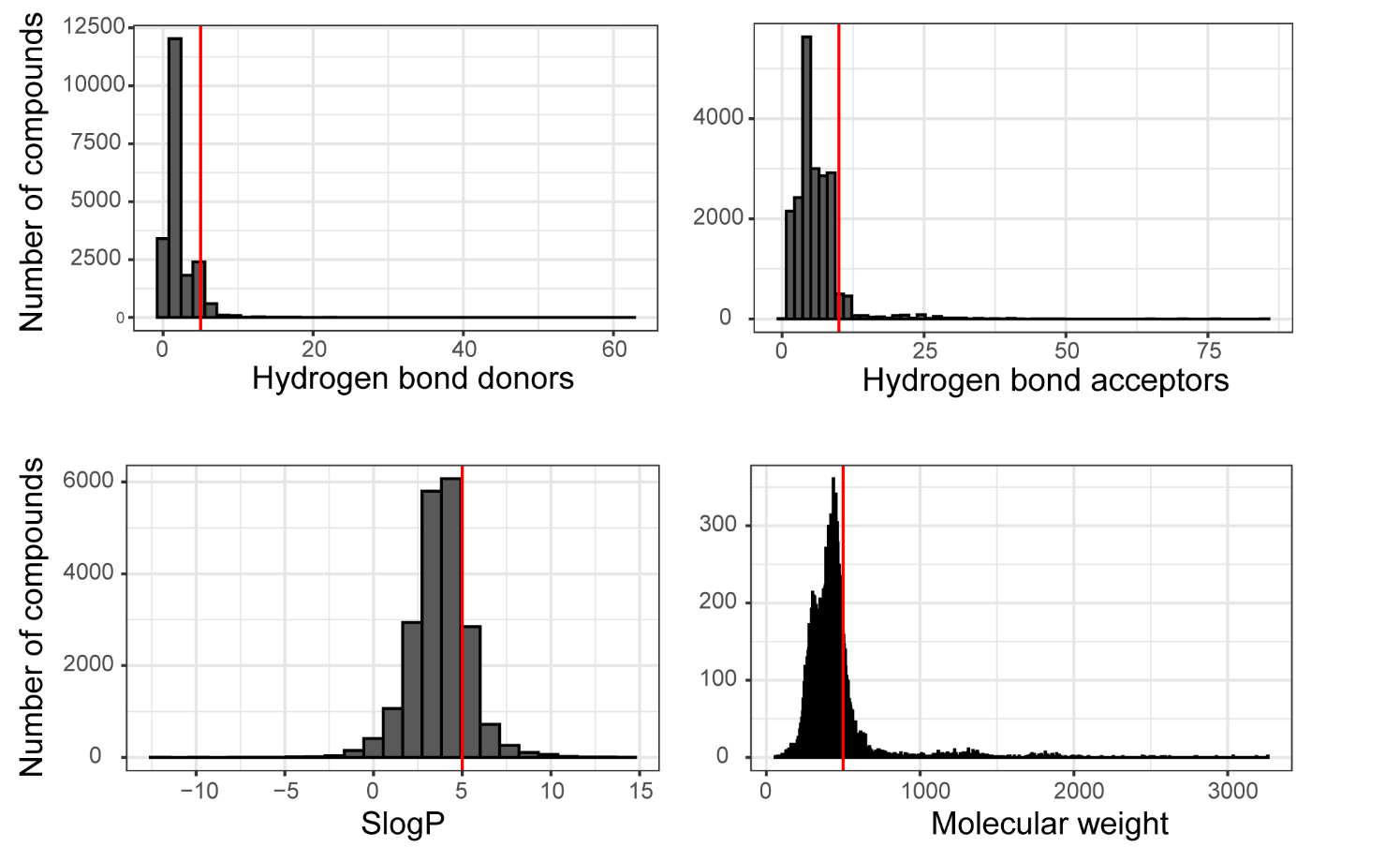


# Supplementary Figure 2. Overview of physicochemical properties of compounds, red line shows Lipinski Rule of 5 five cut offs.


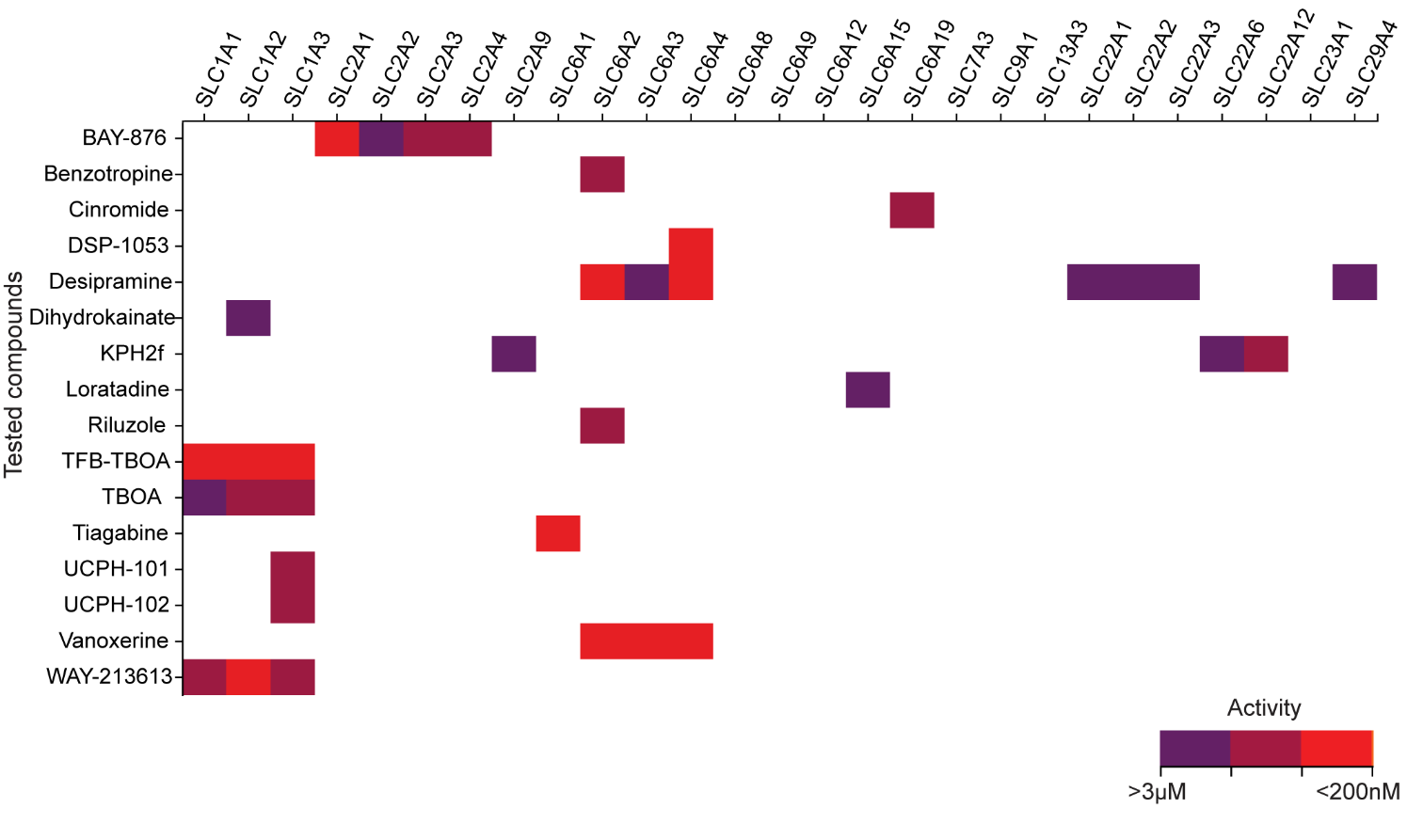


**Supplementary Figure 3.** data availability in the investigated data sources for the compounds that were selected for testing. Violett: activity ≥ 3 µM; brown: 3 µM < activity < 200 nM; red: activity *≤* 200 nM (activity is the median of the reported IC50, Ki, EC50, and Kd values).

**
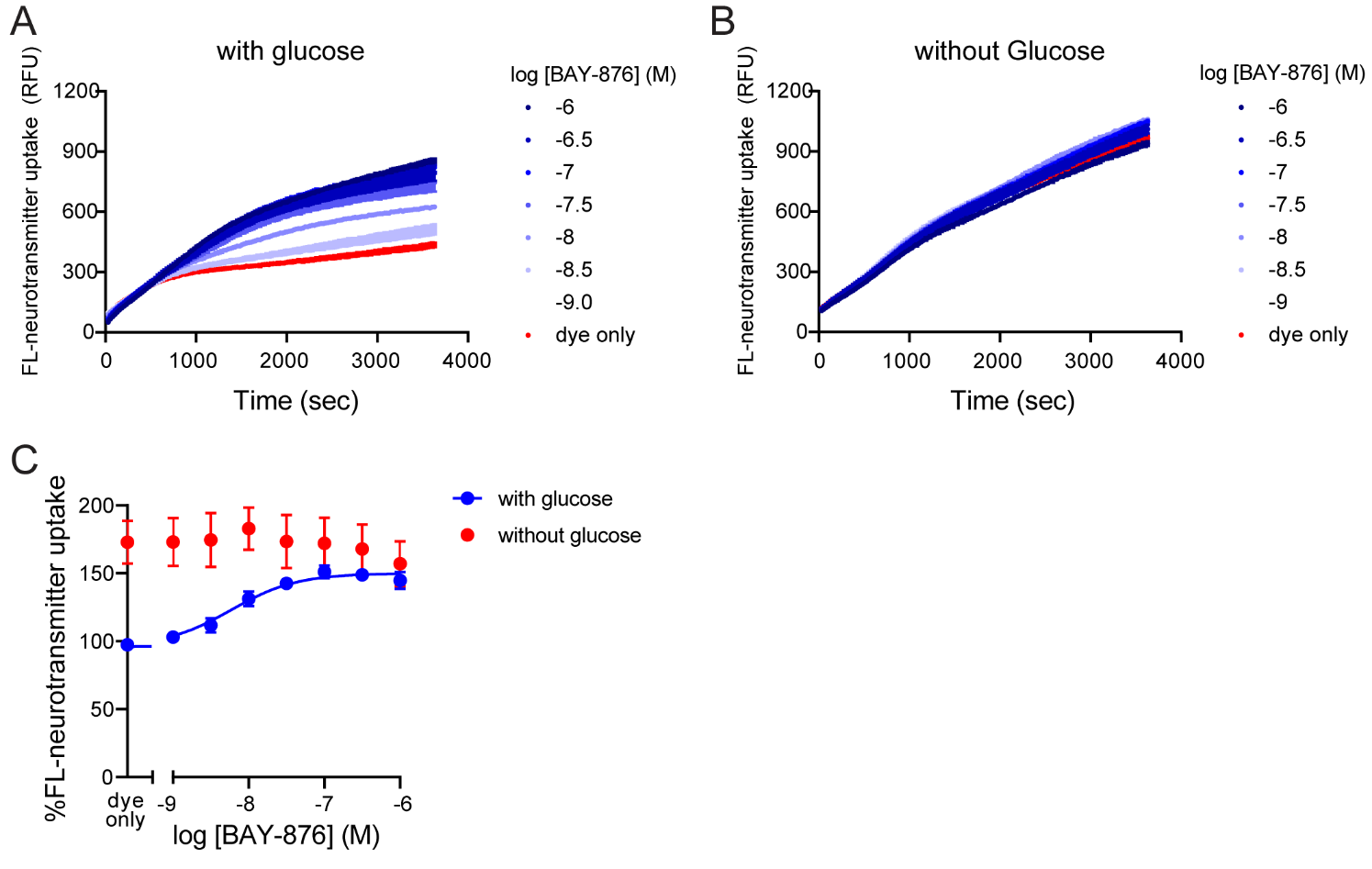
**

**Supplementary Figure 4.** (A) Representative time traces of enhance FL-neurotransmitter uptake by increasing concentration of BAY-876 in HBSS medium with glucose. (B) Representative time traces of FL-neurotransmitter uptake by increasing concentration of BAY-876 in HBSS medium without glucose. (C) Concentration-effect curves of BAY-876 on FL-neurotransmitter uptake in dox-induced HEK-JumpIn-SLC6A4 cells in absence and presence of glucose. Data shown are mean ± SEM of at least 3 experiments performed in duplicate.

**Supplementary Tables**

**Supplementary Table 1.** List of functional assays developed by RESOLUTE (as excel file)

**Supplementary Table 2.** List of compounds targeting SLCs scored (as excel file)

**Supplementary Table 3.** Data for Figure 2C (as csv file)

**Supplementary Table 4.** Data for Figure 2C (as csv file)

**Supplementary Table 5.** List of compounds tested in RESOLUTE assays

| **Name/Synonyms** | **Target family reported** | **Stock conc.** | **Solvent** | **ChEMBL ID** | **Vendor** | **Order-no** | **CAS-no** |
| --- | --- | --- | --- | --- | --- | --- | --- |
| DIDS | SLC4, SLC12, SLC17, SLC26, SLC46 | 10 mM | H2O | CHEMBL1162148 | Sigma | 309795 | 53005-05-3 |
| TFB-TBOA | SLC1 | 10 mM | DMSO | CHEMBL1257519 | Tocris | 2532 | 480439-73-4 |
| Threo-beta-Benzyloxyaspartic acid (TBOA) | SLC1 | 10 mM | DMSO | CHEMBL475341 | Tocris | 1223 | 205309-81-5 |
| Riluzole | SLC1 | 10 mM | DMSO | CHEMBL744 | Sigma | 557324 | 1744-22-5 |
| Dihydrokainate | SLC1 | 10 mM | H2O | CHEMBL279561 | Sigma | D1064 | 52497-36-6 |
| Kainic acid | SLC1 | 10 mM | H2O | CHEMBL275040 | Sigma | 420318 | 487-79-6 |
| WAY-213613 | SLC1 | 10 mM | DMSO | CHEMBL1628669 | Tocris | 2652 | 868359-05-1 |
| UCPH-101 | SLC1 | 10 mM | DMSO | CHEMBL474133 | Tocris | 3490 | 1118460-77-7 |
| UCPH-102 | SLC1 | 10 mM | DMSO | CHEMBL1259233 | Sigma | SML1799 | 1229591-56-3 |
| 1-Methyl-DL-tryptophan | SLC6 | 10 mM | DMSO | CHEMBL513172 | Sigma | M8377 | 153-91-3 |
| Loratadine | SLC6 | 10 mM | DMSO | CHEMBL998 | Sigma | L9664 | 79794-75-5 |
| Cinromide | SLC6 | 10 mM | DMSO | CHEMBL93233 | Sigma/Biozol | ATC386058996-250MG | 58473-74-8 |
| Benzotropine | SLC6 | 10 mM | DMSO | CHEMBL116590 | Enamine | E300-7386482 | 132-17-2 |
| Desipramine | SLC6 | 10 mM | H2O | CHEMBL72 | Tocris | 3067 | 58-28-6 |
| Vanoxerine (GBR12909) | SLC6 | 10 mM | DMSO | CHEMBL281594 | ApexBio | B3249 | 67469-69-6 |
| DSP-1053 | SLC6 | 10 mM | DMSO | CHEMBL4226281 | AstaTech | C92315 | 1176326-76-3 |
| Tiagabine | SLC6 | 10 mM | DMSO | CHEMBL1027 | Sigma | TA9491598258 | 115103-54-3 |
| BAY-876 | SLC2 | 10 mM | DMSO | CHEMBL4448899 | Sigma | SML1774 | 1799753-84-6 |
| KPH2f | SLC22 | 10 mM | DMSO | CHEMBL5222644 | Sanofi | RA18417740A | 2760615-09-4 |

**Supplementary Table 6.** Results of the screening of reported and potential tool compounds, including known data from the KNIME workflow (as excel file)
